# The New Medical Practice Act

**Published:** 1901-03

**Authors:** 


### THE NEW MEDICAL PRACTICE ACT.

We reproduce in this issue the new law concerning the practice of medicine. It passed both houses of the Legislature in the shape it now presents, and was signed by the Governor on February 23rd. By some oversight a part of the emergency clause which would have made the bill operative at once was omitted, and the law can only go into effect ninety days from the day of adjournment of the Legislature;—that is the law. The Legislature will probably not adjourn until the middle of April. That will delay the reform until July or August. It has been asserted and the reporters have extensively circulated the statement, that because the bill stipulates that "the Governor shall appoint the several boards on the 10th day of May following his inauguration," it cannot become effective until the 10th of May, 1903. That is,—the Governor cannot appoint the board prior to the time appointed by the Constitution for the law to take effect, and as next year the 10th of May will not be "immediately following his inauguration"—his successor, the next Governor—will have to appoint the boards on the 10th of May. That is all stuff. A moment's reflection should show any rational person the absurdity of such reasoning. The law is made effective under the Constitution ninety days after adjournment of the Legislature. Unless the Governor appoint the board *at that time* the law *cannot* become operative;—hence his failure to appoint the boards would nullify the law. He must appoint them in order to make the law effective. The Attorney-General has given his opinion, officially, to this effect.

It is much to be regretted that even three or four months must elapse before its benefits will be available, for, in that time the venal and corrupt district boards, the certificate venders, will flood the State with licenses granted to students, and, in fact, to all purchasers; and unfortunately, those licenses, when recorded, are beyond the reach of any law. It is different with diplomas. Bought diplomas, or diplomas from other than "reputable medical colleges"—are to be overhauled by the State Examining Boards, that is,—*all* diplomas that have been registered since 1891—the boards being the sole judges of the character and standing of the colleges granting them. That clause was put in in order to enable the boards to weed out the bogus diplomas. I don't know whether it will work or not,—it looks a little retroactive,—but it is to be hoped that it will. It will matter little after the new law takes effect, how many "medical colleges" spring up, or how low their standard of requirements is,—or how many diplomas they issue or sell,—they will not pass muster, as the holders will not be able to stand the required examination. Hence the once weighty evil of cheap medical colleges is overcome. The effect of strict examinations and a high standard of requirements by the boards will compel colleges to raise their standards and turn out better equipped men or go out of the business. That was the effect the Virginia board had several years ago. It was found that a large number of graduates from certain high character schools could not pass the examination in Virginia, and these colleges realized at once that to keep up their reputation it was indispensably necessary to turn out better educated men.

The new medical law, while it is not all that could be desired—and only partly remedies the evil of "medical anarchy" in Texas—nevertheless has some excellent features, and is a vast improvement on the old one. It will be seen that it gives the medical profession a State Board of Medical Examiners—selected by the State Medical Association, and makes *examination* the test of qualification,—recognizing no diploma, from any source, as a license. There are weak points in it, which, as stated in our last issue, the committee had to accept in order to save the bill in the House. It is hoped that in time these evils may be corrected. It strikes me that one of the greatest dangers—one of the biggest gaps—is that the examining boards for the so-called "other schools" may make merely perfunctory examinations—and license all comers to increase numerically their strength in Texas.

It was argued by the deluded disciples of the "Christian Science" craze,—that to require that "school" to stand a medical examination would be prohibition, as none of those practicing the C. S. methods were educated in medicine. It was claimed that because they give no drugs or medicines they can do no harm. It were useless to argue such a point. Their sin is one of omission—and not of commission. They trust to "faith" or prayer—till it is too late to send for a doctor. But there is no law to compel a man to send for a doctor in any event, and if a man is fool enough to trust his child—sick with diphtheria or other deadly disease—to these cranks,—he has a right, I suppose, to do so,—but it is pretty hard on the child.

But when it comes to the other "schools of medicine" that "do not give or prescribe drugs"—the "drugless doctors", as they call themselves—the line should at least be drawn between "practicing medicine" (without drugs) and practicing surgery. These fellows will tackle anything in which there is a prospect of pay—however small. As illustrating the grave danger from this class, who have unwisely been let loose upon the ignorant people by the Legislature, I will mention an incident which occurred near Austin recently. A "doctor"—who is said to be a "drugless" one—was called to treat a small child, who had a fracture of the neck of the humerus. He said the bone was "outer j'int," and he pulled on it a day or so. The child was brought to Austin and received proper treatment. That man is beyond reach of any reform legislation, or law.

